# Brain Laterality in Dyslexia Seen during Literacy Development and Early Training

**DOI:** 10.3390/brainsci14090893

**Published:** 2024-08-31

**Authors:** Turid Helland

**Affiliations:** Department of Biological and Medical Psychology, University of Bergen, 5020 Bergen, Norway; turid.helland@uib.no

**Keywords:** dyslexia, brain lateralization, dichotic listening, literacy stages, evidence-based training

## Abstract

**Highlights:**

The main finding was that the developmental pattern of brain laterality from the ages of 6 to 11 in the Dyslexia group differed both from the pattern of the Typical group and from a frequently seen pattern in dyslexia.

**Abstract:**

During the period in which children learn to read and write, a gradual shift from right to left hemisphere dominance for language is typically seen. However, in children with dyslexia, a deviant pattern is described in the literature. As part of a larger longitudinal study (The Bergen Longitudinal Dyslexia Study), the present study aimed to assess this development from an early age before children learn to read and write. Dichotic listening (DL), which is a non-invasive test, was used to assess the development of brain laterality in a Typical group and a Dyslexia group. The participants received yearly sessions of evidence-based literacy training at ages 5 to 7. The Typical group showed increasing ear scores and a shift from no ear advantage in the Pre-literacy stage (age 6), indicative of no hemisphere dominance, to a right ear advantage, indicative of a left hemisphere dominance, in the Emergent literacy stage (age 8) and the Literacy stage (age 11). The Dyslexia group showed a different pattern, with a significant right ear advantage at age 6, indicative of a right hemisphere dominance, and increasing ear scores at ages 8 and 11; however, no ear dominance was observed in the Literacy stage. The results point to an effect of relevant, evidence-based training affecting both right and left hemispheres in dyslexia, which should form a basis for further research.

## 1. Introduction

A child’s path to literacy can be separated into three stages [1,2]. The first is the Pre-literacy stage ahead of formalized literacy training in school. Children recognize frequently seen logos but cannot read the individual letters or synthesize them into words. In their play, they often “draw” letters and “read” aloud what they have drawn or “written”. Next is the Emergent literacy stage, when children receive formal literacy training. They are taught the letters, the grapheme/phoneme correspondence, how to synthesize these elements into words, or, reversely, how to analyze the words by their graphemes. The third stage is the Literacy stage. At this stage, reading is automatized, children read whole words or several words at a time, and writing flows easily without analyzing the grapheme/phoneme correspondences of each word. At this stage, literacy is no longer a skill to be learned, but a tool for academic learning.

Although both hemispheres of the brain contribute to language development and learning, the left hemisphere typically becomes dominant during this development [3,4,5]. A gradual shift from right to left hemisphere dominance for language is seen [3]. The contribution of the right hemisphere is less focused in the literature, but it is argued that phonology, writing and speech production affect the hemispheric visual system contributing to a left/right hemisphere interaction in literacy acquisition [6,7,8].

In children with dyslexia, deviant lateralization patterns are often reported. Dyslexia is acknowledged as a specific learning disability, but no consensus is reached on how to define this impairment [9,10,11,12]. The definition by the British Dyslexia Association (BDA) [13] includes the four levels (symptomatic, cognitive, biological and environmental) as described by Morton and Frith [14] and was basic to the present longitudinal study.

According to the BDA definition, at the cognitive level, dyslexia is associated with impaired phonological processing, rapid naming, working memory and processing speed related to functions in the left temporal lobe. Dichotic listening (DL) is a non-invasive method to study brain laterality for language. It has been used for many years to assess functions of the left temporal lobe both for research and clinical purposes. Due to the contralateral neuronal pathways, a right ear advantage in the DL task reflects the superior processing capacity for the right ear stimulus in the left hemisphere [15]. It is considered a reliable and valid assessment tool for language lateralization [16,17], and it has also been shown that DL performance predicts language comprehension skills [18].

The task consists of two different auditory stimuli sent via headphones to both ears simultaneously. The sounds can be monosyllable, nonsense words (consisting of consonant–vowel or consonant–vowel–consonant), numbers, monosyllabic words, rhyme words or words that are closely related semantically [19]. The subject is to report back, usually orally, what he or she heard best. Typically, a right ear advantage is seen, reflecting the fact that the contralateral information (from right ear crossing the right hemisphere back to the left hemisphere) is stronger than the ipsilateral information (from left ear to left hemisphere), with no clear difference between genders [20] and subtle differences in relation to handedness [21].

Traditionally two DL conditions are used for different measurements. Strength is measured by the number of correct responses to stimuli given to each ear. The non-forced condition, where the subject is asked to report what he or she heard best or first, is a measure of language lateralization. The forced conditions, where the subject is instructed to attend to the stimuli given to the right ear (forced right) or the left ear (forced left) is seen as a measure of attention [22].

Lower right ear scores compared to the norm are seen in dyslexia across ages [19]. However, a variety of patterns are reported in dyslexia regarding both the degree of dyslexia severity [23] and the degree of intervention programs offered in school [24]. A severely affected dyslexia group exhibited minor or no right ear asymmetry, while a less affected group showed a right ear advantage in line with controls [24]. Questions have been raised whether DL training could induce a right ear advantage in dyslexia. Studies have shown that short periods of daily training resulted in an increase in right ear scores both in dyslexic and control subjects [25,26]. On the other hand, questions regarding to what degree literacy training affects brain laterality have also been raised [8]. The aim of the present study was to assess the development of brain laterality during the stages of literacy acquisition in a dyslexia group and a typical group. Both groups received extra-curricular literacy training during the Pre-literacy and Emergent literacy stages. Hence, to see if this training could yield new information callosaon the development of brain laterality in dyslexia was also an aim of the study.

## 2. Method

### 2.1. Participants

The DL scores were collected as part of the Bergen Longitudinal Dyslexia Study (also called the “Speak up!” project, https://www.uib.no/en/project/speakup, accessed on 31 August 2024), which has followed a group of children through the three literacy stages. The study was approved by the Regional Committee for Medical Research Ethics in Western Norway (REK-Vest nr.40.03) and the Norwegian Social Science Data Services (NSD).

Assessments were based on the four-level model by Morton and Frith [14] collecting a broad range of data at a symptomatic, cognitive, biological and environmental level. The children were followed closely with yearly testing, three sessions of fMRI assessments and three sessions of literacy training until the age of 11, when dyslexia was identified in accordance with the BDA definition in about 10% of the original participants [27].

A main conclusion from these studies was that a development towards typical scores was seen at the symptomatic, cognitive and biological levels, and that this was mainly explained by the early, evidence-based training the participants received. Specific results are reported in several studies: from the three fMRI sessions [28,29,30,31] and from yearly cognitive assessments [32,33,34]

The participants were children from nine Norwegian preschools selected by the respective county community authorities and with a total of 120 5-year-old children. It should be noted that the Norwegian educational system is inclusive and for all [35], and that all children are followed up by the national health care system for regular health controls [36]. All parents, teachers and clinicians from the preschools were orally informed about the project at local meetings. They were also informed that an at-risk group and a control group formed by fewer than half of the children would be selected to take part in the study. Informed consent was received from 109 parents (90.8%) to let their children participate in a further selection of at-risk children and matched controls. In line with the concept of evidence-based approaches [37] a risk index questionnaire (RI-5) to be filled out by parents and preschool teachers of the 5-year-old children was designed for the purpose to identify early developmental dyslexia risk factors. Notably, a follow-up questionnaire answered by the parents and their children (by then adolescents) 10 years after project start, when the participants were 15 years old, underlined the validity and reliability of the RI-5 for early identification [38,39]. In total, 109 RI-5 questionnaires were sent to and completed by parents and preschool teachers. Criteria of exclusion at project start were impaired sight or hearing, intellectual disability according to DSM-IV criteria [40] and diagnoses of any other impairment included in the DSM-IV (various syndromes such as ADHD, autism spectrum disorders, neurological impairments) as reported by parents. All participants had to have Norwegian as their first language. Based on their RI-5 scores, the 105 children that met the criteria for inclusion were sorted into a risk group (N = 26; M = 13, F = 13) and a matched control group (N = 26; M = 13, F = 13). The remaining children (N = 53) were not followed longitudinally [27].

At project start at age 5, all children were tested with the Wechsler Preschool and Primary Scale of Intelligence™—Third Edition (WPPSI™—III) [27]. There were no significant differences between any of the established groups. Based on the BDA definition, when the children were 11 years old, dyslexia was identified in 13 participants, 11 from the original risk group and 2 from the control group. According to parental reports based on the Children’s Communication Checklist [41,42], a developmental language disorder was identified in five subjects from the Dyslexia group (Dys) when they were 11 years old, i.e. in the Literacy stage [39]. A Typical group (Typ) was formed by the original Control group and the 13 children from the original Risk group. The collected data were then analyzed in retrospect for the Dys and Typ groups. For the present study, valid longitudinal data from dichotic listening tests were collected from 29 Typ subjects (M = 16, F = 13; 5 left-handers) and 13 Dys subjects (M = 5, F = 8; no left-handers). For more information and details on RI-5, see Helland et al. [27]).

Along with yearly assessments, evidence-based training took place every spring for three consecutive years, before school entrance at age 5, then at age 6 (first grade) and age 7 (second grade). The effect of the training is reported by Helland et al. [32]. DL was part of the test batteries when the children were 5, 6, 7, 8 and 11 years old. Due to missing data, ages 5 and 7 were excluded from further analyses.

### 2.2. Tests

Dichotic listening (DL) [43,44] was used to assess the development of brain laterality. This paradigm has shown good reliability values [16,45]. Results from different languages and cultures have shown some differences in DL outcome [46]. In the present study, it was therefore important to keep assessments and comparisons within a Norwegian linguistic context.

Due to the low age of the children at project start, the least demanding DL version from a linguistic point of view, namely, the monosyllable consonant–vowel version, was used. The applied DL test consisted of 36 dichotic stimulus pairs. This included six homonyms, which were used as test trials to assert that the subjects had understood the task but were not included in the statistical analyses. The test was administered in accordance with the manual instructions [47] with the six stop consonants /g/, /k/. /d/, /t/, /p/, /b/ preceding the vowel /a/. Since assessment of language lateralization was the aim of the present study, the non-forced condition was used. The scores were transformed into percentage correct responses for the right ear (Re%) and left ear (Le%).

### 2.3. Data Analyses

Repeated-measures ANOVA was used to assess responses between the factors Group (2: Typ, Dys), Age (3: age 6, age 8, age 11) and Ear (2: Re%, Le%,). For significant effects, Fisher LSD was used as follow-up test.

In addition, t-tests were used in exploratory analyses to assess scores by groups (Typ vs. Dys) and by ear (Re% vs. Le%) separately by the three literacy stages. Since the groups differed as to handedness, a preliminary t-test used in Typ yielded no differences between ear scores in left- and right-handers.

An alpha level was set at *p* ≤ 0.05, and for effect size analyses in the t-tests, Cohen’s d was used (0.2 = small effect, 0.5 = moderate effect, 0.8 = large effect).

## 3. Results

**Figure 1:**
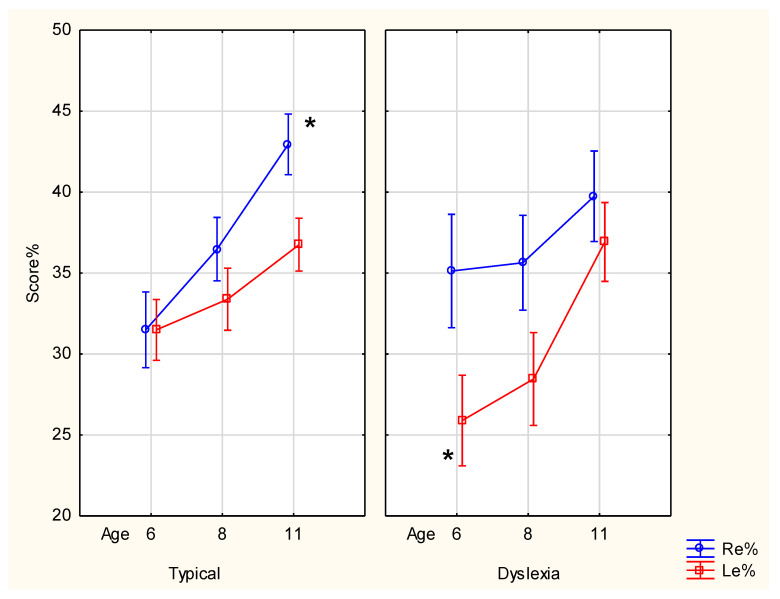
Dichotic listening (DL) scores by groups (Typical: Typ and Dyslexia: Dys) by age and ear, where Re% and Le% represent the percentage correct responses for the right ear and left ear, respectively. Note: * marks significant differences in Typ 11 Re% > all, in Dys 6 Le% < all except Dys 8 Le%.

Repeated-measures ANOVA (Figure 1) showed no triple interaction of Group × Age × Ear (F_2,80_ = 1.508, *p* = 0.227), and no double interactions of Group × Age (F_2,80_ = 0.354, *p* = 0.703), Group × Ear (F_1,40_ = 0.683, *p* = 0.413) or Age × Ear (F_2,80_ = 0.017, *p* = 0.984).

There was no main effect of Group (F_1,40_ = 1.656, *p* = 0.206), but we did observe main effects of Age (F_2,80_ = 25.363, *p* < 0.00001) and Ear (F_1,40_ = 5.61, *p* = 0.023) were significant. An LSD follow-up test showed that the effect of Age was due to significant increases in scores (age 6: 31.19 < age 8: 34.04, *p* = 0.01; age 6: 31.19 < age 11: 39.38, *p* = 0.0001; age 8: 34.04 < age 11: 39.38, *p* = 0.0001). For the Ear scores, a significant effect was due to Re%: 36.93 > Le%: 32.81 (*p* = 0.03).

Within Typ, there were significant effects of Age (age 6: 31.49 < age 8: 34.93, *p* = 0.01; age 6: 31.49 < age 11: 39.85, *p* = 0.0002; age 8: 34.93 < age 11: 39.85, *p* = 0.0003).

In Dys, there were significant effects of Age (age 6: 30.51 < age 11: 38.33, *p* = 0.0001; age 8: 32.05 < age 11: 38.33, *p* = 0.002) and a close to significant effect of Ear (Re%: 36.84 > Le%: 30.43, *p* = 0.06). 

Correct ear responses by groups at the three literacy stages are shown in Table 1.

Typ versus Dys (horizontal analyses in Table 1) yielded no significant group differences at any of the three literacy stages. However, at age 6, Cohen’s d points to Typ Re% < Dys Re% with a large effect size, and Typ Le% > Dys Le% with a moderate effect size. At age 8, Typ Re% = Dys Re% (small effect size) and Typ Le% > Dys Le% with a moderate effect size. At age 11, small effect sizes are seen.

Re% vs. Le% (vertical analyses in Table 1) at each age yielded no significant differences. However, Cohen’s d showed two different developmental patterns. In Typ, a development from little to moderate effect sizes from age 6 to age 11 (Re% > Le%) was seen. In Dys, a development from a moderate effect size at age 6 (Re% < Le%) and a large effect size at age 8 (Re% < Le%) to a small effect size at age 11.

In sum, different developmental patterns were seen in the two groups. Both groups showed increased Re% and Le% by age. Typ developed from no differences in ear scores (Re% = Le%) in the Pre-literacy stage to a larger ear score difference in the Literacy stage. Dys developed from a large difference in ear scores in the Pre-literacy stage to a small difference in the Literacy stage. As illustrated in Figure 1, age 11 Re% in Typ was significantly higher than all other scores in this group. In Dys, age 6 Le% was significantly lower than all other scores in this group except for age 8 Le%. Increases in ear scores were observed in Typ Re% from ages 6 to 8 to 11, and in Dys Le% from ages 6 to 11.

## 4. Discussion

The aim of this study was to assess the development of brain laterality in a typical group and a dyslexia group during the Pre-literacy, Emergent literacy and Literacy stages. The study was part of a larger longitudinal project where extra-curricular evidence-based training was given to the participants in three spring terms covering the Pre- and Emergent literacy stages. Earlier findings from the project were that the Dyslexia group developed a close to normalized performance at the symptomatic and cognitive levels [27,32,33] and at the brain level [28,29,30] by age 11. These positive findings were related to the early training sessions. It was therefore expected that the DL scores would exhibit a similar developmental pattern towards norm scores.

The development of the DL scores of the Dyslexia group differed from the scores of the Typical group. This is in line with reports of brain laterality in language impairments such as developmental language disorder (DLD) and dyslexia [44,48,49]. However, the present data on the DL scores in the Dyslexia group connected to literacy stages and early training offers an alternative pattern of developmental brain asymmetry in dyslexia. To our knowledge, DL scores in preliterate children who received adequate training and were later identified with dyslexia have not been reported before.

### 4.1. Pre-Literacy Stage

Typically, measures of brain laterality for language in illiterates point to no dominance both for children [50] and adults [51]. This aligns with the scores of the Typical group at age 6. However, the Dyslexia group showed substantial differences from this pattern in ear scores, with a right ear dominance at age 6. This is in line with many findings in dyslexia [5,19], notably from older subjects. DL scores from preschool children who later were identified with dyslexia are scarce and comparisons are not feasible [52]. That the Dyslexia group at this stage was behind the Typical group was seen both at brain level assessments [28,29,30] and neurocognitive level [33].

Whether divergent brain laterality in dyslexia is a cause or a function of impaired literacy has been debated for years [53]. The deviant scores in the Dyslexia group at this stage should not be explained at the environmental level alone. In accordance with the Norwegian school system, the children all attended kindergarten and had not been taught literacy yet. The relationship between dyslexia and DLD has long been documented [54,55,56]. However, analyses as to comorbid DLD, to gender differences or effects of handedness were not feasible due to the small sample size of this study but might have contributed to a deeper understanding of the Re% and Le% scores in the Dyslexia group at this stage. Notably, in this study, all children in the Dyslexia group were right-handed, and one may speculate if this connects to the relatively higher Re% over Le% at age 6. Handedness did not seem to affect the scores of the 6-year-olds in the Typical group. 

### 4.2. Emergent Literacy Stage

This stage occurs when children enter school at age 6 and are expected to have “cracked” the literacy code within the first three years of schooling. By the testing at age 8, the children in this study had received three yearly sessions of extra-curricular training in addition to regular school activities [32]. In the Typical group, there was a significant effect of ear compared to the age 6 (Pre-literacy) scores, and in line with what is seen as typical lateralization development. No effect from age 6 to 8 was seen in the scores of the Dyslexia group. Hence, in this respect, one may speculate that the training had a lateralization effect in the Typical group, but not in the Dyslexia group up to this stage. According to one of the brain studies of the Bergen Longitudinal Study, in which only participants from the Typical group took part, a Corpus Callosum refinement process seemed to take place between the ages of 6 and 8. With reference to several dyslexia studies, it was suggested that divergent development as to Corpus Callosum connections and lateralization may be seen in impaired groups such as those with dyslexia [57], which may be one way of understanding the left ear scores in the Dyslexia group at this stage.

### 4.3. Literacy Stage

The Typical group showed an expected and significant right ear dominance in the Literacy stage. That the right ear scores in the Dyslexia group were equal to the right ear scores in the Typical group was not as expected (Reynard et al. [19]). On the other hand, the lack of a difference between the right and left ear scores in the Dyslexia group is often reported in dyslexia [24,25], and was thus as expected.

However, the narrowing of the gap between the Typical group and the Dyslexia group first seen at age 8 and then at age 11 was unexpected. A plausible explanation is that this may be a result of the extra-curricular, evidence-based training that both groups had received through the Pre- and Emergent literacy stages. Strengthening this argument are the positive results from the Bergen Longitudinal Dyslexia Study on structural and functional fMRI scanning [28,29,30], and on specific neurocognitive functions such as language processing [32,33]. Further, literacy scores at age 11 in the Dyslexia group turned out to be within the normal range, albeit at the lower end [27]. The findings are in line with an earlier study showing no difference in DL scores between a dyslexia group and controls where the dyslexia group had been given relevant training. In contrast, the dyslexia group with little or no special training in school showed a significantly lower right ear score and no ear advantage [24]. Also, similar findings have been reported in adult illiterates who became literate after training [51]. 

The significant increase in left ear scores resulted in a minor ear score difference at age 11 in the Dyslexia group. This is in accordance with the earlier reported visual functions of the right hemisphere contributing to a left/right hemisphere interaction in literacy acquisition [6,7,8]. Thus, the present study supports the notion that increased right ear scores are partly a consequence of intervention [48]. 

### 4.4. Limitations of the Study

Firm conclusions cannot be drawn from the present study especially due to the sample size of the Dyslexia group. The developmental pattern of the Typical group was as expected, with no ear dominance in the Pre-literacy stage developing into a right ear dominance in the Emergent literacy and Literacy stages. However, to what extent the variability in the Dyslexia group, especially in the Pre-literacy stage, was an effect of possible DLD, gender and handedness could not be assessed due to the low sample size. It is notable, however, that DLD was identified in five subjects in the Dyslexia group when the participants were 11 years old, i.e. in the Literacy stage [39]. At the start of the project, specific language impairment (SLI) (now DLD) was not diagnosed, but tests of impressive and expressive language were applied, and there was a larger variation in the response patterns within the Dyslexia group compared to the Typical group [32,33,39]. A more valid group identification of early language skills could have contributed to the interpretation of the early DL scores in the Dyslexia group.

### 4.5. Relevance to Practice

A substantial strength of the study is that the participants were followed closely from when they were 5 to 11 years old. The project leaders were in close contact with the participants (children, parents, kindergartens, schools, clinicians, school communities and experienced international researchers) via regular meetings. Group affiliation was never reported to any of the participants. Identification of problems and follow-up was left to regular practices at the participating schools. Within this setting, assessments gained interest and received positive feedback, indicating that DL could or should be included in regular dyslexia assessments. Correctly understood, in line with tests of phonological processing, rapid naming, working memory and processing speed, DL gives meaning both to early diagnosis and intervention. In this context, the DL scores from age 6 should be seen as a valid contribution to early identification.

### 4.6. Further Research

Due to the small number of participants, the present study should be seen as a pilot study. New studies on brain laterality with similar design using the DL paradigm in larger samples distributed by gender and handedness are warranted. In addition to defining 5-year old children at risk by using the RI-5 questionnaire [38], comorbid DLD should be identified. As in the present study, the participants should be followed closely through the three literacy stages until dyslexia can be identified and data analyzed in retrospect.

## 5. Concluding Remarks

The main finding in this study was that the developmental pattern of brain laterality from the ages of 6 to 11 in the Dyslexia group differed from the pattern of the Typical group. The Typical group developed from no ear difference in the Pre-literacy stage to a significant right ear dominance in the Literacy stage, which was an expected and typical development of brain asymmetry. Contrary to this, the Dyslexia group showed a right ear advantage in the Pre-literacy stage and no ear dominance in the Literacy stage. During development, the ear scores in the Dyslexia group increased and showed no significant difference to the Typical group at age 11. These results align with the earlier reported outcomes of narrowing the gap between typical and dyslexia scores in the Bergen Longitudinal Dyslexia Study. However, the developmental pattern in the Dyslexia group was unexpected. One may speculate whether this development was due to the specific training the participants received. Hence, not only should the lateralization aspect be assessed further, but the unexpected increase in left ear scores in dyslexia also needs further attention.

## Figures and Tables

**Table 1:** *t*-test, ear scores in the Typical (Typ) group and the Dyslexia (Dys) group. DL = dichotic listening; n.s. = not significant.

Ear Scores by Stage	Mean Typ, *n* = 29	SD	Mean Dys, *n* = 13	SD	t-Value	*p*	Cohen’s d
Pre-literacy stage							
6 DL Re%	31.50	12.17	35.13	13.58	0.86	n.s.	1.05
6 DL Le%	31.49	9.15	25.90	12.03	–1.66	n.s.	0.53
Re% vs. Le%, t-value, *p*	1.000, n.s		1.387, n.s.				
Cohen’s d	0.00		0.72				
Emergent literacy stage							
8 DL Re%	36.48	11.18	35.64	8.96	–0.24	n.s.	0.08
8 DL Le%	33.39	11.41	28.46	7.15	–1.43	n.s.	0.52
Re% vs. Le%, t-value, *p*	0.964, n.s.		2.116, n.s.				
Cohen’s d	0.27		0.89				
Literacy stage							
11 DL Re%	42.95	10.43	39.74	9.18	–0.95	n.s.	0.33
11 DL Le%	36.75	9.02	36.92	8.22	0.06	n.s.	0.02
Re% vs. Le%, t-value, *p*	1.883, n.s.		0.821. n.s.				
Cohen’s d	0.64		0.32				

Notes. Horizontal analyses: Group (Typ vs. Dys); Vertical analyses: Ear (Re% vs. Le%).

## Data Availability

The original contributions presented in the study are included in the article, further inquiries can be directed to the corresponding author.
